# Role of the granzyme family in rheumatoid arthritis: Current Insights and future perspectives

**DOI:** 10.3389/fimmu.2023.1137918

**Published:** 2023-02-16

**Authors:** Yixin Zheng, Jianan Zhao, Yu Shan, Shicheng Guo, Steven J. Schrodi, Dongyi He

**Affiliations:** ^1^ Department of Rheumatology, Shanghai Guanghua Hospital, Shanghai University of Traditional Chinese Medicine, Shanghai, China; ^2^ Guanghua Clinical Medical College, Shanghai University of Traditional Chinese Medicine, Shanghai, China; ^3^ Institute of Arthritis Research in Integrative Medicine, Shanghai Academy of Traditional Chinese Medicine, Shanghai, China; ^4^ Center for Human Genomics and Precision Medicine, University of Wisconsin-Madison, Madison, WI, United States; ^5^ Department of Medical Genetics, School of Medicine and Public Health, University of Wisconsin-Madison, Madison, WI, United States; ^6^ Arthritis Institute of Integrated Traditional and Western medicine, Shanghai Chinese Medicine Research Institute, Shanghai, China

**Keywords:** rheumatoid arthritis, granzymes, apoptosis, inflammation, biomarker

## Abstract

Rheumatoid arthritis (RA) is a complex autoimmune disease characterized by chronic inflammation that affects synovial tissues of multiple joints. Granzymes (Gzms) are serine proteases that are released into the immune synapse between cytotoxic lymphocytes and target cells. They enter target cells with the help of perforin to induce programmed cell death in inflammatory and tumor cells. Gzms may have a connection with RA. First, increased levels of Gzms have been found in the serum (GzmB), plasma (GzmA, GzmB), synovial fluid (GzmB, GzmM), and synovial tissue (GzmK) of patients with RA. Moreover, Gzms may contribute to inflammation by degrading the extracellular matrix and promoting cytokine release. They are thought to be involved in RA pathogenesis and have the potential to be used as biomarkers for RA diagnosis, although their exact role is yet to be fully elucidated. The purpose of this review was to summarize the current knowledge regarding the possible role of the granzyme family in RA, with the aim of providing a reference for future research on the mechanisms of RA and the development of new therapies.

## Introduction

Rheumatoid arthritis (RA) is a common, long-term autoimmune disease that causes chronic inflammation of synovial tissues in multiple joints. This inflammation can damage cartilage and bone and cause disability (1, 2). RA is more common in women than that in men and occurs at any age (1). Approximately 1% of the population is affected by RA, which significantly affects individuals and society (3, 4). Therefore, it is important to develop novel strategies for timely diagnosis and treatment to reduce inflammation and prevent further damage. Genome-wide association studies have linked the immunopathogenesis of RA to *HLA-DRB1*, a class II major histocompatibility gene (5). Other genes and loci also play a role in the development of RA, including co-stimulatory receptors molecules, cytokine receptor signaling pathways, and activation of the innate immune response (6). Multiple factors, including genetic and epigenetic modifications, immunity, inflammation, microorganisms, metabolism, and other mechanisms, constitute the pathological mechanism responsible for RA in which various immune cells and molecules interact with each other to mediate the autoimmune reaction, eventually causing bone and joint destruction or even disability (7–13).

Despite significant progress in understanding the inflammatory processes involved in RA, the exact mechanism underlying its development and progression is still not fully understood. However, recent studies have suggested that members of the granzyme family play an important role in the immunopathology of RA. Zhang F et al. (14) applied single-cell RNA sequencing, mass cytometry, bulk RNA-sequencing, and flow cytometry to identify the cell populations contributing to joint inflammation in RA. They applied intracellular staining to tissues from RA samples and RNA-seq to sorted CD8 T cells. Intracellular staining of GzmK and GzmB proteins in disaggregated tissue samples from patients with RA revealed that the majority of CD8 T cells in synovial tissue express GzmK. Furthermore, most HLA-DR CD8 T cells express both GzmB and GzmK by intracellular protein staining. Therefore, they defined distinct subsets of CD8 T cells characterized by a GzmK, GzmB phenotype. Defining key cellular subsets and their activation states in the inflamed tissue is a critical step to define new therapeutic targets for RA. Gzms are proteases produced and released by certain immune cells, including cytotoxic T cells (CTLs) and natural killer (NK) cells (15, 16). There are five human Gzms, namely granzyme A (GzmA), granzyme B (GzmB), granzyme H (GzmH), granzyme K (GzmK), and granzyme M (GzmM). Gzms are released into the immune synapse between CLs and target cells, enter target cells with the help of the pore-forming protein perforin, and activate various pro-apoptotic pathways by breaking down intracellular substrates (15, 17). Perforin and granulysin are two pore-forming proteins of cytotoxic granules of human killer cells, and they have significant roles in mediating Gzm responses to infection (18). There’s work showing the role of granulysin as a biomarker and pathogenic factor in RA (19). In addition to playing a role in the process of apoptosis or programmed cell death, Gzms are also involved in the immune response to infection and tissue damage (20, 21). Some studies have shown that Gzms are elevated in the synovial fluid and synovial tissue of patients with RA and may contribute to inflammation and joint damage (22, 23). The known extracellular activities of Gzms suggest a proinflammatory effect in RA. This review aims to summarize the current knowledge on the possible roles of the granzyme family in RA, with the goal of providing a reference for further research into the disease mechanism of RA and the development of targeted therapies.

## GzmA-mediated proinflammatory cytokine-induced bone destruction in RA

Considering all types of killer cells, GzmA is the most abundant Gzm as it is widely expressed in both CD8 CTLs and NK cells (24). GzmA is a serine protease secreted by various CLs, such as NK cells (25), natural killer T (NKT) cells (26), CTLs (27), and CD4 CTLs (28, 29). It plays a key role in the cell death pathway by targeting the endoplasmic reticulum-associated oxidative stress response complex called the SET complex. The SET complex contains at least two GzmA substrates, the nucleosome assembly protein SET (also known as 12PP2A), and the DNA binding protein HMG2. GzmA-mediated cleavage of SET cause inhibition of GzmA-activated DNase NM23-H1 and leads to single-stranded DNA damage (25). GzmA has been shown to have a variety of proinflammatory mechanisms. Hildebrand D et al. (30) suggested that GzmA enters target cells independently and functions as a mediator for inflammation *via* interleukin (IL)-1β cleavage. Wensink AC et al. (31) discovered that treatment of monocytes with GzmA in combination with toll-like receptor-2 (TLR2)- and TLR4-agonists markedly increases the release of proinflammatory cytokines, such as tumor necrosis factor-alpha (TNF-α), IL-6, and IL-8. GzmA also promotes inflammation *via* extracellular activities, such as extracellular cleavage of urokinase (32), proteinase-activated receptor-1 (PAR-1), and PAR-2 (33–36). It is an important proinflammatory mediator in RA (37, 38), psoriasis (39), and osteoarthritis (40). Additionally, NK cells and CTLs transport GzmA into the cytoplasm of target cells through the perforin–granzyme system, and GzmA can cleave the gasdermin B (GSDMB) protein into GSDMB-N and GSDMB-C (at sites K229/K224), releasing its N-terminal pore-forming active fragment, thereby inducing pyroptosis (41).

Abnormal GzmA expression has been linked to inflammatory reactions (23, 42, 43). GzmA and GzmB levels in the plasma and synovial fluid are significantly increased during active periods of RA compared to those during osteoarthritis (OA) (23). GzmA stimulates peripheral blood mononuclear cells to produce TNF-α, IL-6, and IL-8 (36) and stimulates fibroblasts to produce IL-6 and IL-8 (44). These cytokines are largely expressed in the synovium and are mainly produced by macrophages and fibroblast-like synoviocytes (45–47). Therefore, high levels of GzmA in RA joints can promote synovial inflammation owing to its influence on cytokine production. The abnormal expression of GzmA may be related to its abnormal expression in various immune cells, including T cells, NK cells, and NKT cells. Perforin is a 70 kDa glycoprotein that is responsible for the formation of pores on the membrane of target cells (48) and participates in cytotoxic reactions in target cells (49). CD4+ perforin+ and GzmA+ cells have been observed in RA synovial samples (50, 51). Nanki T et al. (52) used flow cytometry to analyze the expression of GzmA and perforin in peripheral blood CD4+ and CD8+T cells of patients with RA and healthy people. GzmA and perforin were mainly expressed by CX3CR1 CD4+ and CD8+T cells in patients with RA and healthy people, with increased expression in patients with RA. In addition, Aggarwal et al. (53) found elevated levels of GzmA in NK and NKT cells and GzmB in NK cells of venous blood samples in patients with RA. Elevated GzmA and GzmB levels are associated with disease severity, tissue damage, and joint damage in RA. Correlation studies showed that the disease activity score (DAS28) is positively associated with enhanced levels of GzmA-expressing NK and NKT cells, perforin-GzmA dual-positive NK, NKT cells, and GzmB-expressing NK cells. Loetscher P et al. (54) analyzed chemokine-mediated enzyme release from cytotoxic lymphocytes using cloned and freshly isolated human blood NK cells and CD8+ T cells. They found that GzmA from CD8+T and NK cells can be activated by chemokines, suggesting chemokines may be involved in regulating cytotoxicity in lymphocyte. Many chemokines capable of inducing Gzms release have been shown to be upregulated in the synovial tissue of patients with RA (55, 56). Therefore, GzmA overexpression in CD8+T and NK cells may be related to chemokines upregulated in RA. Moreover, GzmA and GzmB degrade ECM proteins *in vitro (* 20). Santiago L et al. (37) evaluated inflammatory arthritis induced by type II collagen in wild-type, GzmA-deficient, and perforin-deficient mice, and found that GzmA is more closely associated with cartilage and bone injury in mouse paws and knees than with inflammatory signs and synovial cells. Proliferating osteoclasts (OCs), which are primary bone-resorbing cells, are hematopoietic in origin and have a monocyte/macrophage lineage. The formation and activation of OCs are tightly regulated by systemic and pericellular factors (57). GzmA activates monocytes and other OC precursors to secrete TNF, thus increasing proinflammatory cytokine-induced bone destruction observed in RA. However, the mechanism involved seems to be complex and may be either direct by promoting OC differentiation or indirect *via* other inflammatory responses (37).

## GzmB-mediated inflammation and ECM degradation in RA

GzmB is a granzyme family member with the strongest apoptotic activity because of its caspase-like ability to cleave substrates at aspartic acid residues, thereby activating procaspases directly and cleaving downstream caspase substrates (58). GzmB is a 32 kDa serine protease that is secreted by NK cells and CTLs (59, 60). When released into the gap between those cells and target cells, GzmB can enter the cytoplasm of target cells in the presence of perforin. Subsequently, apoptosis is induced by cleaving various intracellular substrates (61) associated with DNA maintenance, such as inhibitors of caspase-activated DNase, poly (ADP-ribose) polymerase, DNA-dependent protein kinase, and lamin B (62–65). GzmB can be produced by various immune and non-immune cells, including T and B cell subsets, monocytes/macrophages, mast cells, basophils (66–71), vascular smooth muscle cells, lung cells, keratinocytes, chondrocytes, and various types of cancer cells (70, 72–79). GzmB can also have extracellular functions, including the degradation of ECM components, cytokines, cell receptors, and clotting proteins (21, 22, 80). The potential pathophysiological consequences of their cleavage constitute the basis for envisaging a crucial proinflammatory role for GzmB in the pathogenesis of inflammatory diseases (81). In the extracellular pathway, direct processing of caspase-3 and caspase-7 by GzmB promotes caspase-mediated degradation of hundreds of protein substrates, resulting in rapid apoptosis (82).

Abnormal expression of GzmB has been observed in the synovial tissues of patients with RA (83). Studies have shown that most CD8T cells in the synovial tissues of patients with RA express GzmK and GzmB proteins (14). Although Gzms are expressed by CTL, only a small percentage of granzyme-positive cells in the synovial membrane are CD8+ and CD4+ T cells, with the majority being NK cells (84). Elevated levels of GzmB have been found in blood and synovial fluid of patients with RA, which may be a result of GzmB release from inflamed joints (23, 84). Tripathy A et al. (85) indicated that RA patients express functional P2X4 and P2X7 receptors on peripheral CD8+T cells which when ligate with ATP produce high amounts of GzmB. When the ATP molecules induce purinergic signaling and activate T cells *via* P2X receptors (86), the excess extracellular ATP acts as a self-adjuvant to generate abnormal immune responses (87) and triggers inflammation (88, 89). In the case of RA, the release of ATP and its downstream binding to the purinergic receptors is a key regulator of the inflammatory activity (90, 91). The CD8+T cells from RA patients released significant amounts of GzmB in comparison to the CD8+T cells from HCs when stimulated with extracellular ATP. Moreover, the CD8+T cells from RA patients were increasingly activated over time and hence released greater concentrations of GzmB. GzmB is a specific activation marker protein for CD8+T cells. It thus implies that the excess extracellular ATP in the plasma of RA patients can activate immune cells rapidly and hence can be afflictive for the patients.

Goldbach-Mansky R et al. (92) explored the diagnostic and prognostic value of serum GzmB in patients with a diverse spectrum of early inflammatory arthritis and found that GzmB concentrations were significantly higher in rheumatoid factor positive (RF +) RA than those in RF-RA. Patients with joint erosions had significantly higher levels of GzmB than those without, indicating the independent value of GzmB in the prediction of erosive disease. GZMB+CD4 and CD8 CTL cells have also been found to be upregulated in the peripheral blood of active patients with RA (93), potentially reflecting an autoimmune response. Elevated levels of GzmB in blood may result from extracellular GzmB not taken up by the receptor during the induction of apoptotic cell death (92).

GzmB is a multifunctional proinflammatory molecule (94). It can process and activate proinflammatory, pro-fibrotic, and senescence mediators belonging to the IL-1 cytokine family (95, 96). GzmB can process IL-1α into potent proinflammatory fragments, enhancing inflammation. It stimulates interstitial collagenase production by fibroblasts and ECM remodeling, thereby regulating both, normal and aberrant tissue repair (96, 97). Among proinflammatory cytokines, IL-1α/β and TNF-α can trigger the intracellular molecular signaling pathway responsible for RA pathogenesis, which activates mesenchymal cells and synoviocytes and recruits innate and adaptive immune system cells. Synoviocytes, in turn, activate various mediators, including TNF-α, IL-1, IL-6, and IL-8, resulting in synovium inflammation, increased angiogenesis, and decreased lymphangiogensis (98). Therefore, GzmB may be involved in the inflammatory response of RA by regulating IL-1α expression. The role of GzmB in bone destruction in RA has also been suggested by other studies. Single nucleotide polymorphisms in the GzmB gene have been found to influence the joint destruction rate of RA (99). H. K. Ronday et al. (100)found that GzmB can degrade proteoglycan components in cartilage and contribute to the destruction of articular cartilage in RA. Additionally, GzmB is a potential biomarker for RA diagnosis, with higher levels of GzmB in serum being correlated with increased disease activity as measured by the DAS28-CRP score (101). In summary, GzmB may contribute to inflammation and joint destruction associated with RA through its proinflammatory and tissue-degrading effects.

While several studies have reported the role of GzmB as a proinflammatory molecule in the progression of RA proinflammatory, Xu et al. (102) found the frequency of GzmB production by regulatory B cells (Bregs) in patients with RA to be significantly reduced compared to that in healthy controls. The expression of IL-21 receptor in B cells in patients with RA was also significantly reduced, which may contribute to the reduction in GzmB-producing Bregs in these patients. Further analysis showed that the number of GzmB-producing Bregs was negatively correlated with erythrocyte sedimentation rate tender joint count, and disease activity score DAS28. The number of GzmB-producing Bregs increased significantly after RA treatment. A reduction in Bregs, especially those that produce IL-10 has been shown to be negatively correlated with disease activity in RA (103). Those cells may help maintain immune balance by inhibiting proinflammatory cytokine production and T cell differentiation (104). Whether GzmB has cell-specific functional differences remains to be determined.

GzmH and GzmB are structurally similar with 71% amino acid identity and belong to a gene cluster located on chromosome 14, which also includes cathepsin G and mast cell chymase. Although they have high sequence homology, these enzymes have distinct enzymatic activities (105). GzmH has not been detected in NKT cells, monocytes, or neutrophils (106). High levels of human GzmH mRNA have been found in the peripheral blood lymphocytes, lungs, spleen, and thymus (107, 108). Hou et al. (109) discovered that GzmH can induce rapid apoptosis in target cells, resulting in mitochondrial damage, nuclear condensation, and DNA breakage. GzmH-induced apoptosis depends on caspase activation and cytochrome c release. To date, no research has been conducted on GzmH in the context of RA. GzmH is predominantly expressed at high levels in NK cells (110), and GzmH mRNA has also been detected in activated human T cells (107, 111). IL-15 has significantly higher levels in the serum and synovial fluid of patients with RA than those with OA and healthy control groups (112), and plays key roles in promoting activation of NK and CD8 T cells (113). Zhang B et al. (114) stimulated NK-92 cells with IL-15, and it was found that IL-15 significantly up-regulated GzmA and GzmB gene expression, but GzmH transcripts were down-regulated. Therefore, higher levels of IL-15 in patients with RA might regulate Gzms expression. However, the specific role of GzmH in RA requires further investigation, and IL-15 may be a potential target to focus on.

## GzmK- and GzmM-mediated cytokine-based inflammation in RA

GzmK is a trypsin-like molecule in the granzyme family that is expressed by CTLs, NKT, γδ T cells, and CD56bright+ NK cells (110, 115–117). Besides being a member of the granzyme family, little is known about the function of GzmK (118). *In vitro* studies have demonstrated that GzmK can induce non-apoptotic cell death through the production of reactive oxygen species (ROS) and mitochondrial dysfunction when combined with perforin (119). Further studies have shown that GzmK activates caspase-independent apoptosis by cleaving the SET complex, leading to SET destruction. This results in unleashing GzmA-activated DNase NM23H1, which translocates to the nucleus and nicks DNA (120). GzmK may also cleave the tumor suppressor p53, thus sensitizing tumor cells for apoptosis induction (121) and process a vasolin-containing protein, thus contributing to endoplasmic reticulum stress and caspase-independent cytotoxicity (122). GzmK inhibits influenza virus replication in mice (123) and has an immunoregulatory function in multiple sclerosis (124).Cooper DM et al. (125) demonstrated GzmK-induced activation of both ERK1/2 and p38 MAP kinase signaling pathways and significantly increased fibroblast proliferation in patients with sepsis and acute lung inflammation. Wensink AC et al. (126) demonstrated that extracellular GzmK potentiates the lipopolysaccharide-induced release of inflammatory cytokines from monocytes and that this effect is independent of the catalytic activity of GzmK.

GzmK levels in synovial tissue samples from patients with RA are higher than the levels in those with OA (127). GzmK may have proinflammatory effects and can activate PAR-1, a family of G protein-coupled receptors that mediate the physiological response to serine proteases (125, 128). PAR-1 is activated by thrombin and trypsin and can induce the production of inflammatory cytokines, such as TNF-α, IL-1, IL-6, and monocyte chemotactic protein 1 (129, 130). CD8T cells primarily release GzmK, whereas CD4 T cells primarily release GzmB (131). In the context of RA synovium inflammation, GzmK can act as a key inflammatory agent, inducing synovial fibroblasts to activate proinflammatory pathways, including IL-6, CCL2, and ROS production. This effect does not require perforin or any other agent to induce internalization of GzmK, indicating that GzmK has a proteolytic target on the surface of these cells (129). The protease activity of GzmK can also promote degradation of the ECM, leading to inflammatory cell infiltration and tissue destruction. Blocking GzmK or cytokines that activate CD8T cells, such as IL-12 or IL-15, may be an effective treatment for RA. Anti-citrullinated protein antibody-negative (ACPA-negative) RA comprises up to one-third of patients with RA, whereas lack of biomarkers in ACPA-negative RA poses a big challenge to early diagnosis (132). Lu J et al. (133) reintegrated across the GSE89408 dataset to evaluate the performance of GzmK in the diagnosis of ACPA-negative RA. The expression levels of GzmK in the ACPA-negative RA group were significantly higher than that in the normal and OA groups, and the area under the curve of GzmK expression level was 0.916, suggesting its potential as a biomarker.

GzmM is a trypsin-folding serine protease found specifically in the granules of NK cells (134). High levels of GzmM protein and mRNA have been detected in NK, NKT, γδT, and CD8+T cells (118). Studies have shown that human GzmM promotes cell death in a manner similar to GzmB, including caspase-3 activation, DNA fragmentation, ROS production, and the mitochondrial release of cytochrome c (135, 136). Cytoskeletal components, such as α-tubulin and ezrin, nucleolar phosphoprotein nucleophosmin, and apoptosis-associated p21-activated protein kinase 2, have been identified as direct GzmM subunits and are cleaved during GzmM-induced cell death and cytotoxic lymphocyte-induced cell death (137, 138). Synovial fluid-derived mononuclear cells show GzmM expression, with the highest expression in CTLs and NK cells. Elevated levels of GzmM in synovial fluid from patients with RA compared to OA controls have been shown to stimulate human fibroblasts to release IL-29, a proinflammatory cytokine, and type III interferon (IFN-λ1), suggesting that GzmM may play a local role in the pathophysiology of RA (139). Further studies are needed to fully understand the specific role of GzmM in RA.

## Perspectives and challenges

Gzm-inhibiting serpins are believed to act as a fail-safe mechanism for CLs to avoid self-injury during granule exocytosis (140). In recent years, the prevailing theory has been that although circulating Gzms might not be able to enter cells without a high local perforin concentration to induce cell death, they could proteolyze cell surface receptors or extracellular proteins to cause destruction. Particularly when Gzms present at high concentrations at inflamed sites in the absence of natural inhibitors (24). To date, SERPINB12, SERPINB9, SERPINB4, SERPINB1, and inter-alpha inhibitor proteins, have been identified as intracellular inhibitors of GzmA, GzmB, GzmM, GzmH, and GzmK (141–146). Although physiological inhibitors of Gzms are known, no clinical trials have been reported for their use as treatments. Researchers believe that the development of GzmA inhibitors for the treatment of RA may have beneficial effects compared to other commonly used anti-inflammatory drugs, such as corticosteroids or TNF blockers (37). Some studies have suggested that cyclosporine and zidovudine may be potential target drugs for RA treatment in combination with GzmA (43). Zidovudine was developed as an anti-cancer agent in the 1960s and was later approved by the US FDA as an anti-HIV therapeutic drug in the late 1980s after fast track clinical trials (147). Nowadays, this drug is commonly used in the prevention of perinatal HIV-1 transmission (vertical transmission) that consists of the use of this drug by the mother before and during delivery, and treatment of the newborn (148). New potential inhibitors of GzmB, such as tannic acid, mupirocin, cefpiramide, xenazoic acid, vidarabine and phytonadiol sodium diphosphate, have been identified (149). Mi-Sun Kim.et al (150) developed a novel class of weak small-molecule inhibitors against human GzmB by docking studies employing binding site hot spots and three constraints (hydrogen bonding with Arg226, and hydrophobic interactions for S2 and S4 subsites) based on computational solvent mapping using FTMAP. The most distinctive compounds identified were thiazolidinediones 8 (IC50 = 25 μM) and 9 (IC50 = 28 μM), triazole 6 (IC50 = 44 μM), and diazolidinedione 7 (IC50 = 44 μM). Ikram S et al. (151) identified 12 potential inhibitors of GzmH from two separate databases of small molecules. Currently, there are no Gzms inhibitors that are specifically approved for the treatment of RA. Understanding of the precise mechanisms by which granzymes contribute to the development and progression of RA is limited. Lack of clear evidence demonstrating that targeting granzymes is a viable therapeutic strategy for RA. RA is a complex and heterogeneous disease, and it is not clear whether the same granzyme-mediated mechanisms are involved in all patients with RA. There is currently no single, reliable biomarker to indicate patterns of Gzms in patients with RA. Given the intracellular, extracellular, and proinflammatory effects of Gzms on RA, Gzms and their physiological inhibitors may be potential therapeutic targets for RA treatment. It is worth noting that extracellular vesicles (EVs), as one of the important communication carriers between cells and host, may also be a potential contact media between the Gzms family and RA (152). EVs containing granzyme from NK cells and CTLs require Ca2+-dependent signals to release (153). The EVs from activated NK cells include a variety of Gzms, such as GzmA and GzmB, which have cytotoxic effects on tumor cells (154), inhibit cell proliferation, and promote cell death. They are considered a safe and effective immunosuppressive agent, which may have potential therapeutic significance for RA FLS (154, 155).

## Discussion

**Figure 1:**
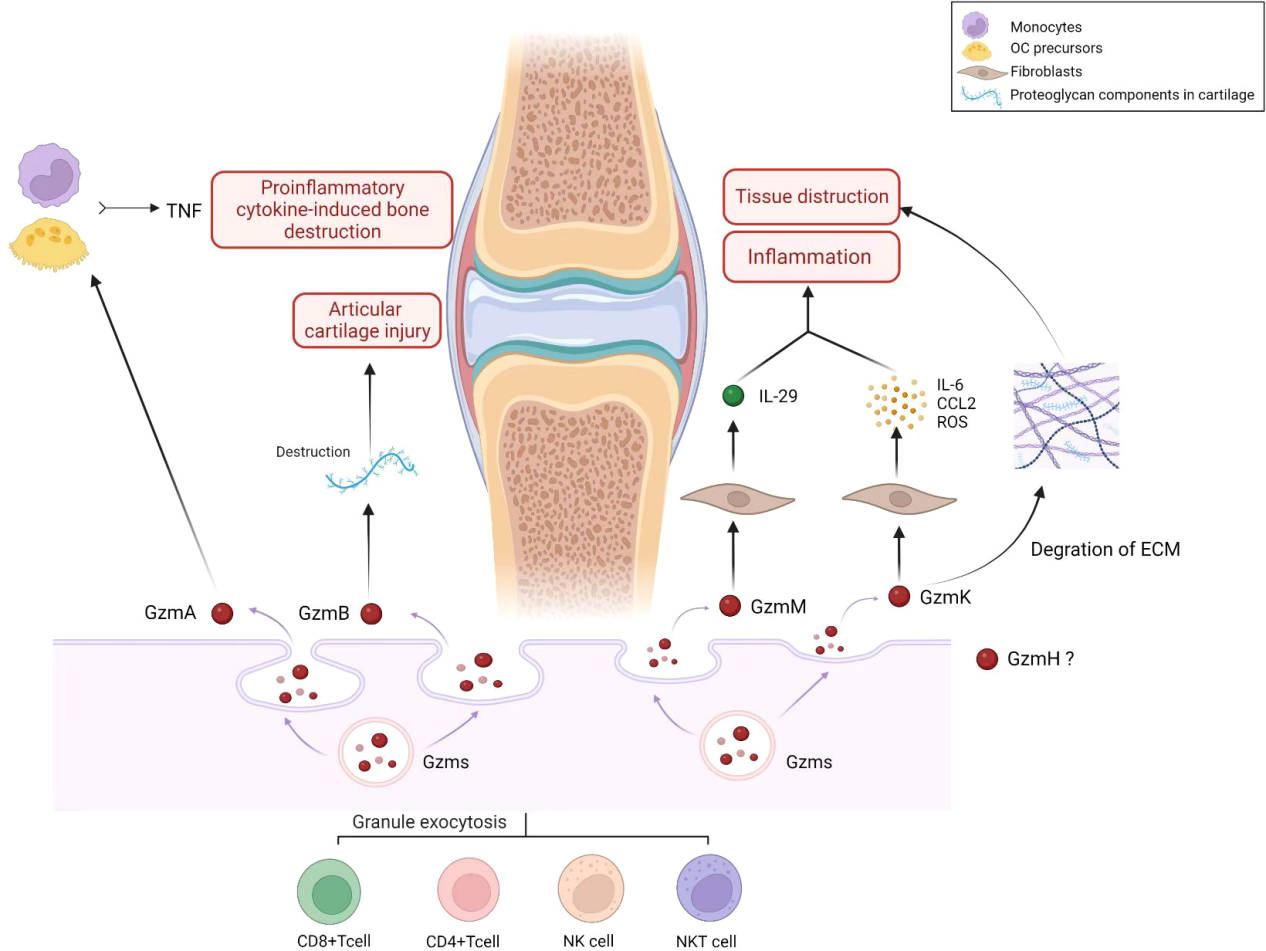
Role of Granzyme family (GzmA, GzmB, GzmH, GzmM, GzmK) in pathology and progression of RA. Gzms are produced and released by immune cells, such as cytotoxic T cells and natural killer cells. They play a role in the ability of the immune system to recognize and eliminate infected or damaged cells. The granzyme family includes several granzyme types, including granzyme A (GzmA), granzyme B (GzmB), granzyme H (GzmH), granzyme M (GzmM), and granzyme K (GzmK). In the context of RA, Gzms may contribute to the pathology and progression of the disease in several ways. RA is an autoimmune disorder characterized by chronic inflammation of the joints that leads to joint damage and deformity. Gzms may contribute to the inflammation and joint damage observed in RA by inducing programmed cell death (apoptosis) in cells within the joint tissue. This can lead to the destruction of joint cartilage and bone, resulting in joint deformity and loss of function. As the figure shows, GzmA activates monocytes and other OC precursors to secrete TNF, thus increasing proinflammatory cytokine-induced bone destruction observed in RA. GzmB can degrade proteoglycan components in cartilage and contribute to the destruction of articular cartilage in RA. GzmM plays a role in the pathophysiology of RA by stimulating the release of proinflammatory cytokine IL-29. GzmK induces synovial fibroblasts to activate proinflammatory pathways, including IL-6, CCL2, and ROS production. The protease activity of GzmK can also promote degradation of the ECM, leading to inflammatory cell infiltration and tissue destruction. (Created with BioRender.com).

In this review, we described the physiological function, cellular expression, and potential role of five members of the Gzms family in RA (**Table 1**). Gzms are involved in the induction of apoptotic cell death. In RA, Gzms demonstrate non-cytotoxic activities that include diverse biological effects, such as stimulation of proinflammatory cytokines and remodeling of extracellular matrices. Considering the extracellular and intracellular functions of Gzms, they have the potential to contribute to the pathogenesis of inflammatory diseases (**Figure 1**). First, GzmA level is significantly elevated in plasma and synovial fluid and can degrade ECM proteins, potentially contributing to bone destruction in RA. Higher levels of GzmB in serum are correlated with increased disease activity. GzmB can degrade proteoglycan components in cartilage and contribute to the destruction of articular cartilage in RA. A subgroup of B cells, Bregs that express GzmB, may inhibit proinflammatory cytokine production and abnormal autoimmune T cell differentiation in patients with RA. GzmM promotes inflammation mainly by stimulating the release of the proinflammatory cytokine IL-29 and is elevated in RA. GzmK is mainly associated with endothelial cells and fibroblasts, suggesting its role in abnormal angiogenesis and synovial hyperplasia in RA. However, the specific role of GzmH in RA requires further investigation. Our search for the latest clinical trials showed that few clinical inhibitors of Gzms have been identified. While the development of clinical drugs targeting the Gzms family is limited, evidence suggests that targeting these proteins may have potential value for the clinical treatment and management of RA. To further enhance our understanding of Gzms in RA, comprehensive use of molecular biology, cellular immunology, and other technologies is necessary. Notably, Gzms primarily play a biological role in cell perforation and target cells. The multiple potential roles of Gzms in RA may include an abnormal manifestation of uncontrolled or excessive cell death. Additionally, the known extracellular activities of Gzms suggest a proinflammatory effect in RA. Therefore, further research on the association between multiple cell death pathways and RA, and experiments defining Gzm-activated proinflammatory pathways may be a promising direction to determine the significance of Gzms as a proinflammatory mediator in future studies.

**Table 1:** The physiological function, cellular expression, and potential role of the Gzms family in RA.

Granzyme	Cellular expression in RA	Granzyme relevance to RA
GzmA	CD4+T cell CD8+T cell NK cell NKT cell	1. Elevation of GzmA in NK and NKT cells associated with disease severity, tissue damage, and joint damage in RA. 2. GzmA activates monocytes and other OC precursors to secrete TNF, thus increasing proinflammatory cytokine-induced bone destruction observed in RA.
GzmB	CD4+T cell CD8+T cell NK cell	1. GzmB concentrations in RF+ RA are significantly higher than those in RF- RA, and patients with joint erosions have significantly higher levels of GzmB than those without, indicating the independent value of GzmB in the prediction of erosive disease. 2. GzmB can degrade proteoglycan components in cartilage and contribute to the destruction of articular cartilage in RA. 3. Higher levels of GzmB in serum are correlated with increased disease activity as measured by the DAS28-CRP score. 4. The expression of IL-21 receptor on B cells of patients with RA is significantly decreased, which may be a possible mechanism of reducing GzmB-producing Breg in patients with RA. Regulatory B cells (Bregs), particularly IL-10-producing Bregs, have been shown to be reduced in number and negatively correlated with disease activity in RA and may contribute to the maintenance of immune functions by inhibiting proinflammatory cytokine production and T cell differentiation.
GzmM	CD8+T cell NK cell	GzmM plays a role in the pathophysiology of RA by stimulating the release of proinflammatory cytokine IL-29, a type III interferon cytokine also known as IFN-λ1.
GzmK	CD8+T cell	In the case of synovial inflammation in RA, GzmK itself acts as a key inflammatory agent. GzmK induces synovial fibroblasts to activate proinflammatory pathways, including IL-6, CCL2, and ROS production. The protease activity of GzmK can also promote degradation of the ECM, leading to inflammatory cell infiltration and tissue destruction.
GzmH	The specific role of GzmH in RA requires further investigation.	

## Author contributions

YZ and JZ is responsible for the collection, collation, and writing of the original manuscript. YS is responsible for the collection. SG, SS, and DH are responsible for the concept development, revision, and manuscript review. All authors contributed to the article and approved the submitted version.
